# Comparative study on liposomal amphotericin B and other therapies in the treatment of mucosal leishmaniasis: A 15-year retrospective cohort study

**DOI:** 10.1371/journal.pone.0218786

**Published:** 2019-06-26

**Authors:** Carolina Rocio Santos, Felipe Francisco Tuon, Juliette Cieslinski, Regina Maia de Souza, Rui Imamura, Valdir Sabbaga Amato

**Affiliations:** 1 Departamento de Molestias Infecciosas e Parasitarias, Faculdade de Medicina, Universidade de Sao Paulo, São Paulo, São Paulo, Brasil; 2 School of Medicine, Pontificia Universidade Catolica do Parana, Curitiba, Parana, Brasil; 3 Laboratório de Parasitologia LIM-46, Instituto de Medicina Tropical, Hospital das Clinicas HCFMUSP, Faculdade de Medicina, Universidade de Sao Paulo, Sao Paulo, Sao Paulo, Brasil; 4 Departamento de Otorrinolaringologia, Hospital das Clinicas HCFMUSP, Faculdade de Medicina, Universidade de Sao Paulo, Sao Paulo, São Paulo, Brasil; Ohio State University, UNITED STATES

## Abstract

**Background:**

Liposomal amphotericin B (L-AMB) has been used for mucosal leishmaniasis (ML), but comparative studies on L-AMB and other drugs used for the treatment of ML have not been conducted. The present study aimed to evaluate the outcome of patients with ML who were treated with L-AMB.

**Methods:**

This is a 15-year retrospective study of Brazilian patients with a confirmed diagnosis of ML. The therapeutic options for the treatment of ML consisted of L-AMB, amphotericin B lipid complex (ABLC), deoxycholate amphotericin B (d-AMB), itraconazole, antimonial pentavalent, or pentamidine. Healing, cure rate and adverse effects (AEs) associated with the drugs used to treat this condition were analyzed.

**Results:**

In 71 patients, a total of 105 treatments were evaluated. The outcome of the treatment with each drug was compared, and results showed that L-AMB was superior to other therapeutic regimens (P = 0.001; odds ratio [OR] = 4.84; 95% confidence interval [CI] = 1.78–13.17). d-AMB had worse AEs than other treatment regimens (P = 0.001, OR = 0.09; 95% CI = 0.09–0.43). Approximately 66% of the patients presented with AEs during ML treatment. Although L-AMB was less nephrotoxic than d-AMB, it was associated with acute kidney injury compared with other drugs (P <0.05).

**Conclusion:**

L-AMB was more effective than other therapies for the treatment of ML. However, a high incidence of toxicity was associated with its use. Therapeutic choices should be reassessed, and the development of new drugs is necessary for the treatment of ML.

## Introduction

The tegumentary form of the leishmaniasis can be classified in two main clinical forms: cutaneous and mucosal. Mucosal leishmaniasis (ML) is primarily caused by *Leishmania braziliensis* (*L*. *braziliensis*), and it occurs months or years after the cutaneous lesions have healed [1]. Mucosal leishmaniasis is a progressive disease that can destroy cartilages and the osseous structures of the face, pharynx, and larynx [2]. Most ML cases are treated with antimonial pentavalent. However, this drug has several adverse effects (AEs) and contraindications [3]. Liposomal amphotericin B (L-AMB) is a safe option. However, the cost of this drug can be a limitation in developing countries, and the ideal dose is not established.

L-AMB is the first-line treatment for visceral leishmaniasis [4], and it has been used for mucocutaneous leishmaniasis with controversial results. In some studies, the healing rate was similar to those who were not on therapy [5]. The variability of clinical response to L-AMB is associated with the *Leishmania* species. In Brazil, studies have found different cure rates [6, 7].

Considering the controversial benefits of L-AMB on the treatment of ML, the present study aimed to evaluate the treatment outcome of the largest cohort of patients with ML who were treated with liposomal amphotericin in comparison with other treatments.

## Methods

This is a retrospective study that included a cohort of patients with a confirmed diagnosis of ML who received at least one treatment with any leishmanicidal drugs from January 2000 to July 2015. The study was approved by the local ethical committee (CAPpesq number: 0576/11). Informed consent was waived by the ethics committee considering the characteristics of this study. All data were fully anonymized before access. Patients from the different cities of Brazil were treated in a reference hospital in Sao Paulo, SP, Brazil.

The inclusion criteria were as follows: patients with confirmed ML who were older than 18 years and followed-up for at least 6 months. Exclusion criteria included patients without confirmation of ML and refuse to treatment. The diagnosis of ML consisted with the identification of *Leishmania* spp. in the tissue via molecular tests, culture, or the observation of the typical structure during histological examination or immunohistochemistry [8]. Otorhinolaryngological examinations were performed by the same physician throughout the study period.

The clinical variables included in the analysis were gender, age, Brazilian state of origin, race, previous cutaneous lesions suggesting cutaneous leishmaniasis, comorbidities, duration of the symptoms, site of the ML, symptoms, serum and cutaneous tests, and tomographic and clinical findings, as previously classified [9, 10].

The therapeutic options for the treatment of ML consisted of L-AMB (1–4 mg/kg/day), amphotericin B lipid complex (ABLC) (1–4 mg/kg/day), deoxycholate amphotericin B (d-AMB) (1 mg/kg/day), itraconazole (200 mg/day for 6 weeks), antimonial pentavalent (20 mg/Sb^+5^/kg/day for 30 days), or pentamidine (4 mg/kg for 10 days). All formulations of amphotericin B should achieve a cumulative dose of 2,500 mg. Antimonial pentavalent was considered the drug of first choice. In the case of contraindications, the second choice drugs used were pentamidine and amphotericin B. For those cases where these therapeutic options were used without success or presented contraindication, lipid formulations of amphotericin B has been used. For refractory cases or contraindication to all therapeutic regimens, itraconazole was used.

Cure was defined as the total healing of mucosal lesion based on otorhinolaryngological and fiberoptic examinations until 1 year after the end of the therapy. Failure is defined as the absence of lesion improvement after therapy or the return of lesions before 1 year. Lesions that showed an improvement after 6 months of therapy but did not completely heal were considered failure. Recurrence is considered as new lesions or the return of the lesion after 1 year of therapy [11].

In addition, the AEs associated with the treatment of ML were analyzed during the hospitalization period: fever, headache, nausea or vomiting, tremors or chills, sweating, phlebitis on the infusion site, low back pain, chest pain, palpitation, myalgia, arthralgia, asthenia, and rashes. Systemic AEs included acute kidney injury (AKI) according to the AKIN criteria [12], electrolyte imbalance, hepatic enzyme alterations, pancreatic enzyme alterations, myelotoxicity, electrocardiographic changes, and hypoglycemia. Changes in hepatic and pancreatic enzymes were considered when their values exceeded three times the baseline value or, in the absence of baseline values, when they exceeded three times the normal limit. Myelotoxicity was considered when hemoglobin level decreases by 2 points from baseline, total leukocyte level is below 3,000/mm^3^, and/or platelet count is below 150,000/mm^3^.

Data of the patients were organized as general data, and the risk factors of the treatment were evaluated. New cases included patients who received more than one treatment during follow-up. Thus, in 71 patients, a total of 105 treatments were analyzed. The number of treatments exceeded the number of patients because one patient could be followed more than one treatment. A successful treatment was considered when achieved complete healing within 3 months. The secondary treatment followed a protocol using the different chemotherapy schedule.

Continuous data were expressed as mean or median with standard deviation (SD) or ranges. Frequencies were expressed as percentages. Categorical, continuous, and dichotomized independent variables were analyzed with forward conditional factorial binary logistic regression model to determine the statistical significance of the clinical and epidemiological findings, diagnosis, and treatment along with the outcome and recurrence of ML. A P-value <0.05 was considered statistically significant. All data were recorded in Microsoft Excel (Microsoft), and statistical analysis was performed using SPSS version 23.

## Results

In 71 patients, a total of 105 treatments were evaluated. All patients have a confirmed diagnosis of ML. The mean age of the patients was 59.9 years, and approximately 61% of the study participants were men. Most patients came to our service with symptoms for more than 5 years (65.4%). The most common symptoms were epistaxis, nasal obstruction, and rhinorrhea. The nasal mucosa was the more commonly affected site in leishmaniasis (75.2%), followed by the pharynx and palate (Table 1). Additional data are available in the S1 File.

**Table 1:** Clinical and laboratorial findings of 71 patients with mucosal leishmaniasis.

Data		N = 71	%		
Brazilian Region					
	Northeast	36	50.7		
	Southeast	21	29.6		
	South	6	8.4		
	North	2	2.9		
	Midwest	6	8.4		
Sex					
	Female	27	38.0		
	Male	44	62.0		
Age (years±SD)		59.1±14.2			
Comorbidities					
	Systemic arterial hypertension	44	62.0		
	Diabetes mellitus	7	9.9		
Previous cutaneous lesions		37	52.1		
	Inferior Limbs	22	31.0		
Duration of symptoms					
	<1 year	12	17.0		
	1–5 years	16	22.5		
	5–10 years	30	42.2		
	>10years	13	18.3		
Symptoms					
	Epistaxis	48	68.5		
	Nasal obstruction	50	71.4		
	Rhinorrhea	38	54.2		
	Nasal crust	26	36.6		
	Pruritus	16	22.5		
	Cacosmia	14	19.7		
	Sneeze	4	5.6		
	Odynophagia	9	12.7		
	Hyposmia	6	8.5		
	Facial pain	10	14.1		
Local of the mucosal leishmaniasis					
	Nasal	55	78.5		
	Palate	14	19.7		
	Pharynx	18	25.4		
	Larynx	10	14.2		
Nasofibroscopy					
	Septal perfuration	47	66.2		
	Granulomatous activity	38	54.3		
	Crusts	45	64.2		
	Edema	12	17.1		
	Stenosis	14	20.0		
	Bleeding	4	5.7		
	Mucosal atrophy	5	7.1		
	Hyperemia	13	18.6		
	Ulceration	4	5.7		
	Purulent drainage	2	2.8		
Diagnosis					
	Histological exam	70	98.5		
	Positive immunohistochemistry	20	28.5		
	Polymerase chain reaction (14 patients)	10	71.4		
Serum test					
		ELISA*		IIF**	
	1:40	16	27.5	12	16.9
	1:80	5	7.0	7	9.8
	1:160	3	4.2	2	2.8
	1:320	9	12.6	0	0.0
	>1:320	7	9.8	2	2.8
	Reagent without titre	4	5.6	3	4.2
	Non reagent	5	7.0	18	25.3
	Positive Montenegro test	41	57.7		
CT scan findings (n = 48)					
	Thickening of mucosa	18	37.5		
	Sinusopathy	10	20.8		
	Mastoideopathy	4	14.3		
Hospitalization for treatment		57	80.3		
	Duration of admission (days±SD)	22.7±10.5			
	Treatment interrupted by side effect	31	43.6		
Follow up duration (months±SD)		55.6±46.6			
Outcome					
	Cure	37	51.1		
	Relapse	12	16.9		
	Interruption by side effect	31	43.6		
Final cure		46	64.8		

* ELISA (enzyme linked immunosorbent assay)

**IIF—indirect immunofluorescence

Most patients reported a previous cutaneous lesion (52.4%), which was located mainly in the lower limbs. Moreover, 9 patients reported previous lesions in the upper limbs. One patient reported a lesion in the back, and another patient had a lesion in the penis. In addition, one patient reported a lesion in the face.

All patients were subjected to nasofibroscopy. Septal perforation was identified in 66.2% of the participants, followed by crusts and granulomatous inflammation of the mucosa. Computed tomography (CT) scan of the face has been included in the routine examination of ML since 2009. In 48 patients who were evaluated, 70.8% presented with mucosal thickening. Other findings are detailed in the Table 1.

The treatment of all patients with ML was performed in the hospital with a mean hospitalization time of 22.9 days. In the treatment of these patients, L-AMB was the most commonly used drug (32), followed by pentavalent antimonial (25), d-AMB (14), ABLC (13), pentamidine (11), and itraconazole (10). The treatment outcome of each drug was compared, and results showed that L-AMB was superior than other therapeutic regimens (P = 0.001; odds ratio [OR] = 4.84; 95% confidence interval [CI]: 1.78–13.17). The risk factor associated with the healing was the absence of mucosal thickening on CT scan (Table 2). From 105 treatments, healing was achieved in 61, but cure rate was only 64.8%. In the multivariable analysis, L-AMB was an independent variable associated with healing (P = 0.008), and absence of mucosal thickness on CT scan too (P = 0.038). The group of patients that received L-AMB was clinically similar with other treatments, except by stenosis in nasopharinx on nasofibroscopy exam (Table 3).

**Table 2:** Comparative data of patients with mucosal leishmaniasis with complete healing after treatment and without healing.

		Healing		No Healing			
Data		N (61)	%	N (44)	%	P Value	OR 95%CI
Sex							
	Female	23	37.7	18	40.9	0.448	
	Male	38	62.3	26	59.1		
Age (years±SD)		60.2±14.3		60.0±12.3		0.935	
Comorbidities							
	Systemic arterial hypertension	38	62.3	26	59.1	0.448	
	Diabetes mellitus	5	8.2	6	13.6	0.534	
Previous cutaneous lesions		34	55.7	21	47.7	0.611	
Duration of symptoms							
	<1 year	7	15.9	5	13.5	0.835	
	1–5 years	9	20.5	7	18.9		
	5–10 years	16	36.4	14	37.8		
	>10years	12	27.3	11	29.7		
Symptoms							
	Epistaxis	41	65.6	33	75.0	0.283	
	Nasal obstruction	44	70.5	31	70.5	0.478	
	Rhinorrhea	33	54.1	21	47.7	0.266	
	Nasal crust	23	37.7	23	52.3	0.127	
	Pruritus	16	26.2	10	22.7	0.393	
	Cacosmia	12	19.7	9	20.5	0.589	
	Sneeze	6	9.8	3	6.8	0.410	
	Odynophagia	6	9.8	8	18.2	0.188	
	Hyposmia	6	9.8	7	15.9	0.283	
	Facial pain	8	11.5	5	11.4	0.596	
Local of the mucosal leishmaniasis							
	Nasal	46	75.4	33	75.0	0.569	
	Palate	9	14.8	13	29.5	0.061	
	Pharynx	14	23.0	16	36.4	0.110	
	Larynx	7	11.5	5	11.4	0.608	
Nasofibroscopy							
	Septal perfuration	39	63.9	32	72.7	0.248	
	Granulomatous activity	31	50.8	20	45.5	0.344	
	Crusts	39	63.9	26	59.1	0.353	
	Edema	8	13.1	8	18.2	0.336	
	Stenosis	10	16.4	11	25.0	0.372	
	Bleeding	2	3.3	2	4.5	0.564	
	Mucosal atrophy	3	4.9	6	13.6	0.115	
	Hyperemia	9	14.8	11	25.0	0.158	
	Ulceration	2	3.3	5	11.4	0.110	
	Purulent drainage	1	1.6	2	4.5	0.382	
CT scan findings (n = 48)							
	Thickening of mucosa	16	26.2	18	40.9	0.044	0.24 [0.05–1.02]
	Sinusopathy	8	13.1	5	11.4	0.454	
	Mastoideopathy	2	3.3	3	6.8	0.379	
Therapy***							
	L-AMB	26	81.3	6	18.7	0.001	4.84 [1.78–13.17]
	d-AMB	2	13.3	12	86.7	0.001	0.09 [0.01–0.43]
	ABLC	6	46.1	7	53.9	0.272	0.58 [0.18–1.88]
	Antimonial pentavalent	14	56.0	11	44.0	0.567	1.03 [0.41–2.60]
	Itraconazole	4	40.0	6	60.0	0.196	0.45 [0.12–1.71]
	Pentamidine	8	72.7	3	27.3	0.231	2.10 [0.52–8.43]

*** Percentages for therapy are related with patients treated with each drug

L-AMB—liposomal amphotericin; ABLC—amphotericin B lipid complex; d-AMB—desoxycholate amphotericin B

**Table 3:** Comparative data of patients with mucosal leishmaniasis treated with -AMB and those treated with other options (itraconazole, pentamidine, d-AMB, ABLC, antimonial pentavalent).

		Other		L-AMB		
Data		N (73)	%	N (32)	%	P Value
Sex						
	Female	31	42.5	10	31.3	0.194
	Male	42	57.5	22	68.7	
Age (years±SD)		61.8±13.7		59.1±13.5		0.366
Comorbidities						
	Systemic arterial hypertension	46	63.0	18	56.3	0.330
	Diabetes mellitus	8	11.0	3	9.4	0.555
Previous cutaneous lesions		37	82.2	18	78.3	0.464
Duration of symptoms						
	<1 year	8	14.5	4	15.4	0.877
	1–5 years	11	20.0	5	19.2	
	5–10 years	19	34.5	11	42.3	
	>10years	17	31.0	6	23.1	
Symptoms						
	Epistaxis	51	69.9	23	74.2	0.422
	Nasal obstruction	51	69.9	24	77.4	0.296
	Rhinorrhea	39	53.4	15	48.4	0.399
	Nasal crust	30	41.1	16	51.6	0.220
	Pruritus	18	24.7	8	25.8	0.542
	Cacosmia	12	16.4	9	29.0	0.117
	Sneeze	5	5.5	5	16.1	0.087
	Odynophagia	10	13.7	4	12.9	0.593
	Hyposmia	8	11.1	5	16.1	0.342
	Facial pain	10	13.7	3	9.7	0.417
Local of the mucosal leishmaniasis						
	Nasal	54	69.0	25	68.8	0.576
	Palate	16	21.9	6	18.8	0.466
	Pharynx	20	27.4	10	31.3	0.428
	Larynx	9	12.3	3	9.4	0.472
Nasofibroscopy						
	Septal perfuration	49	63.9	22	72.7	0.248
	Granulomatous activity	35	49.3	17	53.1	0.442
	Crusts	48	67.6	17	53.1	0.118
	Edema	10	14.1	7	21.9	0.239
	Stenosis	10	14.1	11	34.4	0.020
	Bleeding	0	0.0	4	12.5	*
	Mucosal atrophy	7	10.0	2	6.3	0.420
	Hyperemia	15	21.4	6	18.8	0.489
	Ulceration	5	7.0	2	6.3	0.624
	Purulent drainage	1	1.4	2	6.3	0.227
CT scan findings (n = 48)						
	Thickening of mucosa	22	71.0	12	70.6	0.614
	Sinusopathy	8	25.8	5	29.4	0.522
	Mastoideopathy	3	9.7	2	11.8	0.588

L-AMB—liposomal amphotericin B

* cell with value = 0

The AEs associated with the treatment of this condition were analyzed. Approximately 66% of the patients presented with AEs during ML treatment. The most frequent symptoms were infusion-related AEs (fever, chills, sweating, and palpitations) in 48.1% of the participants, followed by phlebitis (20.2%) and nausea/vomiting (18.3%). The most common systemic AEs were electrolyte imbalance (28.8%) and AKI (19.2%) (Table 4). d-AMB had worse AEs than other regimens (P = 0.001; OR = 0.09; 95% CI = 0.09–0.43). ABLC was associated with infusion-related AEs (P<0.05). Meanwhile, antimonial pentavalent was associated with metabolic disturbances, such as hyperamylasemia and increased liver enzymes. However, fewer electrolyte disorders were observed when amphotericin is used (P<0.05). Although L-AMB was less nephrotoxic than d-AMB, it was associated with AKI compared with other drugs (P<0.05).

**Table 4:** Adverse effects of drugs used in the treatment of mucosal leishmaniasis.

		Global		Antimonial Pentavalent		Pentamidine		ABLC		d-AMB		Itraconazole		L-AMB			
Data		N	%	N	%	N	%	N	%	N	%	N	%	N	%	P value*	P value
Total Adverse events (AE)		79	76.0														
Clinical																	
	Infusion related AE	50	48.1	11	44.0	5	45.5	10	76.9	7	50.0	0	0.0	17	54.8	0.026	
	Phlebitis	21	20.2	2	8.0	4	36.4	6	46.2	3	21.4	0	0.0	6	19.4	0.023	
	Nausea/vomits	19	18.3	3	12.0	3	27.3	5	38.5	4	28.6	0	0.0	4	12.9		
	Fever	9	8.7	2	8.0	0	0.0	3	23.1	1	7.1	0	0.0	3	9.7		
	Headache	9	8.6	2	8.0	1	9.1	1	7.7	3	21.4	0	0.0	2	6.5		
	Chest pain	8	7.7	1	4.0	0	0.0	3	23.1	0	0.0	0	0.0	4	12.9		
	Tremor	7	6.7	0	0.0	0	0.0	4	30.8	0	0.0	0	0.0	3	9.7	0.004	0.031
	Chills	6	5.8	0	0.0	0	0.0	2	15.4	3	21.4	0	0.0	1	3.2		
	Muscle pain	6	5.7	3	12.0	0	0.0	1	7.7	0	0.0	0	0.0	2	6.5		
	Lmbar pain	5	4.8	0	0.0	0	0.0	1	7.7	0	0.0	0	0.0	4	12.9	0.027	
	Palpitations	5	4.8	1	4.0	0	0.0	1	7.7	0	0.0	0	0.0	3	9.7		
	Arthralgia	4	3.8	4	16.0	0	0.0	0	0.0	0	0.0	0	0.0	0	0.0	0.003	
	Sweat	3	2.9	0	0.0	0	0.0	2	15.4	0	0.0	0	0.0	1	3.2	0.041	
	Gastrintestinal other	3	2.9	3	12.0	0	0.0	0	0.0	0	0.0	0	0.0	0	0.0		
	Malaise	2	1.9	1	4.0	1	9.1	0	0.0	0	0.0	0	0.0	0	0.0		
	Rash	1	1.9	1	4.0	0	0.0	0	0.0	0	0.0	0	0.0	0	0.0		
Laboratorial					0.0												
	Electrolyte disturbance	30	28.8	1	4.0	4	36.4	6	46.2	7	50.0	0	0.0	12	38.7	0.001	0.001
	Acute kidney injury	20	19.2	0	0.0	0	0.0	1	7.7	8	57.1	0	0.0	11	35.5	0.001	0.008
	Liver enzyme alteration	7	6.7	3	12.0	0	0.0	0	0.0	0	0.0	0	0.0	0	0.0	0.042	
	Hyperamylasemia	7	6.7	7	28.0	0	0.0	0	0.0	0	0.0	0	0.0	0	0.0	0.001	
	ECG alterations	5	4.8	5	20.0	0	0.0	0	0.0	0	0.0	0	0.0	0	0.0	0.001	
	Any mielotoxicity	3	2.9	1	4.0	1	9.1	1	7.7	0	0.0	0	0.0	0	0.0		
	Hypoglicemia	1	1.0	0	0.0	1	9.1	0	0.0	0	0.0	0	0.0	0	0.0		

* P value for gray box in the line. The first p value is reference to the first gray box from left to right. The second P value is reference to the second gray box from left to right.

## Discussion

Data on L-AMB have been published in the literature since 1990, and in 1997, the FDA approved its use for visceral leishmaniasis. With regard to the efficacy of the drug in the treatment of cutaneous leishmaniasis, the efficacy rate ranged from 84% to 85% [13–15]. The few published studies on the treatment of ML with L-AMB presented a small number of patients and reported a high efficacy rate between 83.3% and 100% [6, 7, 11]. This study enrolled 32 patients treated with L-AMB and was the first to compare L-AMB with other drugs used in the treatment of ML. The cure rate of the group that used L-AMB was 5 times higher than that of the other groups (OR = 4.89, 95%CI [1.78–13.17], p = 0.001). The group “healing” was different from “non-healing” considering only the thickness of mucosa on CT, a finding that probably cannot influence therapeutic outcome according with different drugs. The group of patients treated with L-AMB was similar with the group of patients treated with other drugs. The number of patients treated with each drug was too small to compare with those treated with L-AMB, so, we grouped the patients treated with itraconazole, pentamidine, d-AMB, antimonial pentavalent and ABLC. L-AMB is the first choice treatment for visceral leishmaniasis [4], and the current study suggest that L-AMB should be considered in the group of first line options.

Our study revealed a therapeutic success rate of 58.3% with antimonial use. Some studies have shown a clinical cure rate between 71.0% and 77.0% of the cases [16, 17]. Recurrence with the use of antimonials is around 22% [2]; our study showed a rate of 15.3%. Pentamidine has an efficacy rate of approximately 90%, and recurrence was observed in 25% of the treated cases [3]. Moreover, pentamidine (72.7%, 8/11 patients) had a greater efficacy than pentavalent antimonial (58.3%, 14/24 patients). However, no statistically significant difference was observed. In this study, pentamidine had fewer adverse events than other drugs. The use of amphotericin B deoxycholate should be limited, although it produces a small number of recurrences and has better action on mucosal lesions compared to antimonials [18]. The number of adverse events was high, and in this study, the actual recurrence rate with the use of amphotericin B deoxycholate cannot be evaluated due to the large number of patients who were not able to complete the treatment.

Studies that compared lipid formulations and the use of ABCL in the treatment of ML are not available. Previous studies on the use of amphotericin B colloidal dispersion in the treatment of ML showed an efficacy rate between 88% and 100% [14]. This is the first study that used ABCL in the treatment of ML, and results showed a success rate limited to 46.2% (6/13) and a relapse rate of 7.7% in patients who were treated with a high rate of permanent interruption (61.5%).

Despite the fact that itraconazole is a safe therapeutic option for the treatment of ML, its use had a low efficacy (44.4%). A recent review of the use of azoles in the treatment of ML revealed a 50% efficacy rate: 37% for ketoconazole and 61% for fluconazole and itraconazole, which varied according to the species of *Leishmania*, with the worst responses to *L*. *braziliensis*, which is the most important species in Brazil [19]. In total, 38 patients with ML were treated with azoles, showing an efficacy rate of 52.6%, ranging from 23% to 73.3% [20, 21].

In Brazil, ML is considered a public health problem due to its morbidity. Most ML cases involved men due to increased work-related exposure to sandflies [22]. In addition, a high level of testosterone increases the production of IL-4 and TGF-β, leading to more severe lesions [23]. The most frequent symptoms of ML were nasal obstruction (72.9%), epistaxis (70.0%), and rhinorrhea (52.9%), as observed in the literature [24]. According to Marsden (1986), these are among the earliest symptoms of ML. However, late diagnosis was an important factor in our study because only 7 out of 50 patients (14%) were diagnosed within the first year of symptom onset and 32 (64%) after 5 years or longer. In the examination of the mucosa, erythema, infiltration, erosion, ulceration, crusts, and mucopurulent exudate can be observed. The most common findings on rhinoscopy and/or nasofibroscopy were septal perforation, crusts, and granulomatous process in 66.2%, 65.2%, and 58.0% of the cases, respectively. Different results were recorded in the literature, and septal perforation was observed in 41.4–77.0% of the patients [11, 25].

The sinus CT of the face revealed alterations in 86.2% of the patients. Mucosal thickening of some paranasal sinuses occurred in 62.1% (18/29) of the cases, and sinusopathy was observed in 27.2% of the participants. Camargo et al. have revealed the expressive value of 96.0% for paranasal sinus mucosal thickening on CT scan, suggesting that inflammation in patients with ML is not restricted to the nasal mucosa and may extend to the paranasal sinuses and other structures of the respiratory tract [10].

In our study, more than 70% of the patients presented with some AEs. The drugs with more AEs were d-AMB and ABCL. Pentavalent antimonial had presented electrocardiographic and pancreatic enzyme changes occurred in 20% and 28% of the patients who used the drug, respectively (p = 0.005; p <0.001). In addition, these AEs were the primarily cause for the permanent interruption in the use of this drug (8 out of 11 interruptions). The most frequent pentamidine AE was phlebitis (36.4%), followed by nausea and vomiting (27.3%). Despite the high frequency rate of the general AEs in this study, the use of pentamidine is considered safe, as previously described [11]. The use of ABCL led to a significantly high incidence rate of phlebitis (46.2%) and tremors (30.8%) compared to other leishmanicidal drugs (p = 0.029 and p = 0.005). The AEs of ABLC are more common than those of L-AMB [26]. These observations have important clinical relevance because the infusion in 8 of the 13 patients who used ABLC was interrupted. Nevertheless, the liposome of amphotericin B attenuates the release of cytokines, which decreases infusion-related AEs [27]. In contrast, the rapid removal of ABCL from the circulation by reticuloendothelial tissues, particularly in the liver [28], may result in the release of proinflammatory cytokines from the surrounding macrophages and contribute to the AEs associated with the administration of ABCL.

The major limitations of this study are the retrospective analysis, few patients for sub-analysis, different duration of the disease. Unfortunately these biases cannot be controlled. The doses of drugs were different among patients with the same therapeutic group, considering that this was not a clinical trial.

This retrospective study suggests that L-AMB is an effective drug in the treatment of ML. Approach of patients with ML should be reconsidered and L-AMB could be considered as a first line of therapy. A controlled study is need in this context, but at this moment, this is the best evidence we have to treat ML. The treatment of ML treatment is still far from ideal because the best-acting drugs for this condition have numerous AEs, and drugs that are safer to use have a low efficacy. L-AMB presented with better results than other drugs used for the treatment of ML. However, the incidence rate of nephrotoxicity associated with this drug is high.

## Supporting information

S1 File Clinica data of patients. (XLSX)
